# Mitochondrial Dysfunction in Periodontitis and Associated Systemic Diseases: Implications for Pathomechanisms and Therapeutic Strategies

**DOI:** 10.3390/ijms25021024

**Published:** 2024-01-13

**Authors:** Yifei Deng, Junhong Xiao, Li Ma, Chuan Wang, Xiaoxuan Wang, Xin Huang, Zhengguo Cao

**Affiliations:** 1 State Key Laboratory of Oral & Maxillofacial Reconstruction and Regeneration, Key Laboratory of Oral Biomedicine Ministry of Education, Hubei Key Laboratory of Stomatology, School & Hospital of Stomatology, Wuhan University, Wuhan 430079, China; yifeideng@whu.edu.cn (Y.D.);; 2 Department of Periodontology, School & Hospital of Stomatology, Wuhan University, Wuhan 430079, China

**Keywords:** mitochondria dysfunction, mitochondrial quality control, oxidative stress, periodontitis, periodontology, dentistry, systemic disease, therapy

## Abstract

Periodontitis is a chronic infectious disorder damaging periodontal tissues, including the gingiva, periodontal ligament, cementum, and alveolar bone. It arises from the complex interplay between pathogenic oral bacteria and host immune response. Contrary to the previous view of “energy factories”, mitochondria have recently been recognized as semi-autonomous organelles that fine-tune cell survival, death, metabolism, and other functions. Under physiological conditions, periodontal tissue cells participate in dynamic processes, including differentiation, mineralization, and regeneration. These fundamental activities depend on properly functioning mitochondria, which play a crucial role through bioenergetics, dynamics, mitophagy, and quality control. However, during the initiation and progression of periodontitis, mitochondrial quality control is compromised due to a range of challenges, such as bacterial–host interactions, inflammation, and oxidative stress. Currently, mounting evidence suggests that mitochondria dysfunction serves as a common pathological mechanism linking periodontitis with systemic conditions like type II diabetes, obesity, and cardiovascular diseases. Therefore, targeting mitochondria to intervene in periodontitis and multiple associated systemic diseases holds great therapeutic potential. This review provides advanced insights into the interplay between mitochondria, periodontitis, and associated systemic diseases. Moreover, we emphasize the significance of diverse therapeutic modulators and signaling pathways that regulate mitochondrial function in periodontal and systemic cells.

## 1. Introduction

Periodontitis is a chronic infectious disease characterized by the progressive destruction of periodontal tissues, including the gingiva, periodontal ligament, alveolar bone, and cementum [1]. This multifactorial disease is initiated by dysbiotic microbial communities in the oral biofilm, triggering an aberrant host immune response [2]. The sustained presence of these pathogens, combined with the host immune response, leads to the release of pro-inflammatory cytokines, matrix metalloproteinases, and reactive oxygen species (ROS), resulting in the degradation of collagen fibers, alveolar bone resorption, and periodontal ligament collapse [3].

Mitochondria are double-membraned organelles found in eukaryotic cells, often described as “energy factories” due to their essential role in energy metabolism through ATP generation. However, mitochondria also play a crucial role in numerous cellular processes, including mitophagy, ROS regulation, and signaling transduction [4]. Recent evidence suggests that mitochondrial dysfunction contributes to the pathogenesis and tissue destruction of periodontitis by inducing oxidative stress (OS), inflammation, and even mitochondria-mediated apoptosis [5]. However, further research is warranted to fully elucidate the intricate mechanisms underlying the involvement of mitochondrial dysfunction in the etiology and progression of periodontitis, while considering the interplay with other contributing factors [6,7]. This review focuses precisely on this aspect, providing a detailed exploration of the interaction between mitochondria and periodontal pathogenic bacteria, as well as the role of mitochondrial dysfunction in the cellular damage of periodontal tissues.

Periodontitis has gained increasing recognition as a potential contributor to the development and progression of various systemic diseases. Epidemiological studies have shown associations between periodontitis and conditions such as cardiovascular diseases, Type II diabetes, and cognitive impairment [8,9,10]. While the exact mechanisms underlying these associations remain unclear, emerging evidence suggests that mitochondrial dysfunction may play a significant role [7,11,12,13,14,15]. Mitochondrial impairment can lead to increased oxidative stress, inflammation, and dysregulated energy metabolism, which are key pathological features observed in both periodontitis and these systemic diseases [7]. Understanding the involvement of mitochondrial dysfunction in the pathogenesis of systemic diseases associated with periodontitis may provide valuable insights for the development of novel therapeutic approaches targeting both oral and systemic health.

## 2. Mitochondrial Quality Control and Dysfunction

### 2.1. Mitochondrial Quality Control System

Mitochondria have been regarded as the centerpiece organelle of eukaryotic cell energy metabolism for decades [16]. Yet, with the exploration of the mitochondrial quality control (MQC) system, its deeper functions have been gradually uncovered. As an integrated network that monitors mitochondrial homeostasis, MQC coordinates various processes such as mitochondrial dynamics, mitophagy, and mitochondrial biogenesis [17].

#### 2.1.1. Mitochondrial Dynamics

Mitochondrial dynamics are the most fundamental form of quality control and a prerequisite for other functions. While carrying out heavy bioenergetic activities, mitochondria face various environmental changes and pressures within and outside the cell, leading to gradual deterioration of individual mitochondrial function over time. Mitochondria alleviate this burden and increase efficiency by fusing with each other and sharing internal materials [18]. Mitochondrial dynamics refer to the dynamic balance between fusion and fission [16], which influence mitochondrial morphology, function, and quantity. Mitochondrial fission is primarily mediated by Drp1 and Fis1, while the fusion process involves mitochondrial outer membrane (OMM) fusion regulated by Mfn1/2 and mitochondrial inner membrane (IMM) fusion regulated by OPA1 [18,19].

#### 2.1.2. Mitophagy

When this burden is further aggravated, mitochondria can divide and reject damaged parts resulting from the aging process, and then, with the help of autophagy, decompose the remains into recyclable material [20]. The occurrence of mitophagy starts with the loss of normal mitochondrial membrane potential (MMP), the accumulation of the mitochondrial outer membrane kinase PINK1, and the recruitment of the E3 ubiquitin ligase Parkin from the cytoplasm. Under the ubiquitination of Parkin, mitochondria are engulfed by the separation membrane and fuse with lysosomes [21]. The inhibition or abnormal enhancement of mitophagy by various stimuli may lead to cell dysfunction or even death.

#### 2.1.3. Mitochondrial Biogenesis

Mitochondria cannot be synthesized de novo, but can only maintain their function through a process of alternation between old and new. PGC-1α is a key regulator in mitochondrial biogenesis, which promotes TFAM synthesis and mitochondrial DNA transcription by activating Nrf1/2 [18,22]. Multiple antioxidants promote mitochondrial function and protect cells by upregulating PGC-1α [23,24,25,26]. Mitochondrial membrane dynamics, mitophagy, and mitochondrial biogenesis are the three main modes of mitochondrial quality control.

### 2.2. Mitochondrial Dysfunction

Mitochondrial dysfunction arises due to external or internal stress, leading to impaired MQC system reflected by reduced biosynthesis, disrupted dynamics, dysregulated mitophagy, etc. Additionally, it results in abnormal metabolism, mitochondrial DNA (mtDNA) efflux, oxidative stress, and mitochondria-mediated apoptosis.

#### 2.2.1. MtDNA Efflux

Mitochondria are descendants of α-proteobacteria and became part of present-day eukaryotic cells through endosymbiotic events about 2 billion years ago [7]. This particular prokaryotic origin retains some features for mitochondria. When its components, especially mtDNA, are released into the cytoplasm and systemic circulation due to quality control failure, they have the “antigenicity” of directly activating innate immunity and triggering inflammatory responses in immune sentinel cells [27]. This finding can be traced back as early as 2004, when Collins et al. found that the intra-articular injection of mtDNA in mice caused local inflammation [28]. Recent studies have further revealed that the cytoplasmic release of mtDNA can activate the cGAS-STING pathway, TLR9 pathway and inflammasome formation, thereby upregulating the synthesis and secretion of various pro-inflammatory cytokines, such as IL-1β, IL-18, and IFN [27]. Although the mechanism of mtDNA efflux remains unclear, current research suggests two main possibilities: one involves the formation of mitochondrial outer membrane pores and the extrusion of mitochondrial inner membrane, leading to mtDNA release during cell apoptosis, and the other involves mitochondrial permeability transition pore (MPTP) opening induced by the inflammasome [27].

The proximity of mtDNA to the respiratory chain, which generates high levels of free radicals, renders it more susceptible to oxidative damage compared to nuclear DNA. Oxidized mtDNA (Ox-mtDNA) is particularly prone to triggering the formation of the NLRP3 inflammasome and has been implicated in various diseases such as heart failure and atherosclerosis [27]. In fact, the inflammation-inducing effect of mtDNA may be partly attributed to the oxidized residues within its structure: if only an oligodeoxynucleotide with the same sequence, but no oxidized residues, is injected, there is no inflammatory provocation [28].

In addition to intracellular inflammation, mtDNA can also be released into the extracellular space, such as being packaged into extracellular vesicles (EVs) for genome transport [29]. In addition, the latest studies have found that macrophages can release mitochondrial RNA (mtRNA) to promote cytokine secretion and mediate interferon response [30].

#### 2.2.2. Oxidative Stress

ROS are natural products of cellular oxidative metabolism. Mitochondrial respiration is the main source of endogenous ROS. Under physiological conditions, about 90% of cellular oxygen is consumed by mitochondria, with 1–5% converted to ROS [7]. Superoxide anion free radical (O_2_-) and hydrogen peroxide (H_2_O_2_) are the two primary ROS in the human body. They can arise from enzymatic reactions in mitochondria, ER, and peroxisomes, as well as non-enzymatic processes like nutrition, toxins, and radiation. ROS can damage DNA, proteins, and sugars by reacting with ferrous ions. Additionally, ROS act as signaling molecules in redox pathways (e.g., Nrf2, NF-κB, PGC-1α, p53), regulating immunity, development, and aging [31]. Thus, balancing ROS production and clearance is vital for cellular function.

Oxidative stress refers to the unbalanced state in which more ROS and other oxidants are produced than those cleared in the body, resulting in interrupted REDOX signaling pathways and damaged molecules [32]. As mentioned above, the mitochondrial electron transport chain is responsible for the majority of ROS production; therefore, inefficient oxidative phosphorylation is more prone to induce mitochondria dysfunction, which manifests as the loss of MMP [7].

#### 2.2.3. Mitochondria-Mediated Apoptosis

Mitophagy is a process that helps maintain cellular homeostasis, while programmed cell death occurs when various stimuli surpass the cell’s maintaining capacity. Mitochondrial apoptosis, also known as intrinsic apoptosis, is one of the best characterized pathways in cell death. When DNA damage, oxidative stress, and other stimuli activate pro-apoptotic factors of the Bcl-2 family (primarily Bax and Bak) to induce mitochondrial outer membrane permeability (MOMP). Subsequently, Drp1-mediated ridge remodeling and calcium influx occur, initiating downstream apoptotic signaling cascades (including inner membrane cytochrome c release and subsequent caspase activation), eventually resulting in cell death [33]. In addition, mitochondria also play an influential role in other forms of cell death such as necroptosis, ferroptosis, and pyroptosis [33,34].

## 3. Effects of Periodontal Pathogens and Their Virulence Factors on Mitochondria

Periodontitis is a disease characterized by the chronic infection of periodontal pathogens and inflammatory destruction. At present, it is widely recognized that the key pathogens affecting the state, progression, and treatment effects of periodontitis are as follows: *Porphyromonas gingivalis* (*P. gingivalis*), *Aggregatibacter actinomycetemcomitans* (*A. actinomycetemcomitans*), *Tannerella forsythia* (*T. forsythia*), *Fusobacterium nucleatum* (*F. nucleatum*), etc. [35]. The pathogenic mechanisms of these bacteria still need to be investigated further, but it is already clear that mitochondria are an important target in the interactions between these bacteria and their virulence factors and the host.

Quite a few studies have shown that periodontal pathogen infection has a full range of effects on the structure and function of mitochondria. *P. gingivalis*, and its extracellular vesicles can induce metabolic changes in macrophages, shifting from oxidative phosphorylation to glycolysis [36]. *T. forsythia*-segregating factors accumulate in host mitochondria after infection and inhibit enzyme activity [37]. Moreover, mitochondrial membrane dynamics are also affected. *P. gingivalis* upregulates Drp1 in vascular endothelial cells, promoting mitochondrial fission [11]. Conversely, EVs released by *F. nucleatum* promote mitochondrial fusion and the invasion of colorectal cancer cells [38]. More recently, Aral et al. also showed that *P. gingivalis* and *F. nucleatum* specifically regulate the expression of mitochondria-ER contact-related genes such as MFN1, GRP75, IP3R, and PINK1. These findings enhance our understanding of the host–microbe interactions involved in the pathogenesis of periodontitis. Furthermore, periodontal pathogens can disrupt the MQC system. *P. gingivalis* reduces the expression of mitophagy genes like PINK1 and impairs the clearance of damaged mitochondria, thereby exacerbating the inflammatory response in macrophages during bacterial infection [39].

Lipopolysaccharide (LPS) or endotoxins of Gram-negative bacteria are highly toxic and antigenic to periodontal tissue, playing a significant role in the development of periodontal disease. LPS-induced mitochondrial dysfunction involves increased oxidative stress, impaired energy production, and disrupted biosynthesis [26]. Treatment with *P. gingivalis*-LPS has been observed to affect oxidative phosphorylation, glycolysis and mitochondrial biosynthesis [40,41,42]. *P. gingivalis*-LPS treatment in vitro also reduced mtDNA copy number and caused sustained mtDNA efflux into plasma by opening the MPTP [40]. 

Periodontal pathogens employ various mechanisms to manipulate mitochondria and influence cell death, aiding in host invasion. *P. gingivalis* affects apoptosis in a time-dependent manner. Boisvert et al. found that *P. gingivalis* rapidly manipulates mitochondria to resist apoptosis through the translocation of gingipain adhesion peptide A44 in the early stage of epithelial cell infection (within 12 h), thus resisting host clearance [43]. However, after the early stage of infection (after 24 h), *P. gingivalis* upregulated the apoptosis-inducing factor AIF in epithelial cells, induced caspase-independent mitochondrial apoptosis, and promoted oral tissue damage [44]. Apoptosis induced by periodontal pathogens has also been observed in other systemic diseases associated with periodontitis. *P. gingivalis*-LPS upregulates XBP1 in adipocytes and induces mitochondria-mediated apoptosis [45]. *A. actinomycetemcomitans*-LPS significantly increases the incidence of apoptosis in human placental trophoblast cells [46]. This effect may be critically involved in the pathogenesis of periodontitis-related systemic diseases. Additionally, besides apoptosis, ferroptosis and pyroptosis are implicated in *P. gingivalis*-LPS-induced periodontitis. Ferroptosis-related genes are upregulated in *P. gingivalis*-LPS-treated cells and rat periodontitis models, leading to mitochondrial shrinkage, increased membrane density, and elevated ROS levels [47]. *F. nucleatum* and *P. gingivalis* exosomes activate inflammasomes to induce pyroptosis. However, direct infection by *P. gingivalis* does not activate inflammasomes [36,48]. These facts suggest that the different roles of *P. gingivalis* and its EVs in chronic periodontitis need to be explored.

## 4. The Relationship between Mitochondria and Periodontal Tissue Cells

**Figure 1:**
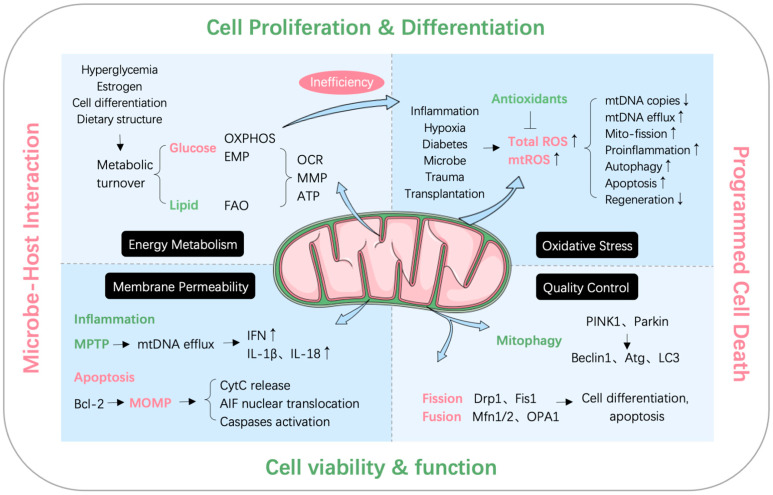
Mechanisms of mitochondria influencing periodontal disease and health status. Pointed arrows: promoting effect; bar-headed arrow: inhibitory effect. The figure was generated using Adobe Photoshop software (version 22.0.0) and Servier Medical Art, provided by Servier, licensed under a Creative Commons Attribution 3.0 unported license.

Mitochondria affect cellular and physical health in numerous ways. In terms of periodontitis, many causative agents for periodontitis induce mitochondrial metabolic turnover, mtDNA efflux, oxidative stress, mitophagy, and altered membrane dynamics [11,40,49,50,51]. Figure 1 summarizes the mechanisms by which mitochondria affect periodontal tissue disease and health status.

The destruction of periodontitis is typical in several types of periodontal tissue cells. In terms of soft tissues, the apoptosis of gingival epithelial cells induces periodontal biological barrier collapse [52,53]. The oxidative stress and inflammatory state of gingival fibroblasts is associated with periodontitis progression [54]. Moreover, periodontal ligament stem cells exhibit impaired osteo-/cementogenic differentiation under pathological conditions [55]. In terms of hard tissues, a disturbed osteoblast–osteoclast balance aggravates alveolar bone resorption [56]. Less noticeable are the impaired generation and mineralization of cementoblasts, which are significant features of periodontitis [22]. These pathological changes are closely related to mitochondrial dysfunction.

This chapter describes the impact of mitochondria dysfunction on various periodontal cells from the perspective of the four periodontal tissues.

### 4.1. Gingiva

#### 4.1.1. Gingival Epithelial Cells

Gingival epithelial cells (GECs) form the barrier of periodontal tissue to resist the invasion of pathogenic microorganisms. Apoptosis and intercellular junction destruction will cause the collapse of epithelial barrier [52]. Additionally, the manipulation of GEC apoptosis by periodontal pathogens directly hinders the clearance of initial infection [53]. Multiple substances and pathogens can cause mitochondria-dependent apoptosis in GECs.

Volatile sulfur compounds and tobacco extract can induce mitochondrial MMP loss and apoptosis in GECs, leading to local epithelial damage [57,58]. The effect of cyclosporine A, an immunosuppressant that induces gingival hyperplasia, on GEC apoptosis is still debated. At the same dose (30 mg/kg) in rats, it has been reported to enhance mitochondrial apoptosis in gingival keratinocytes [59], yet inhibit mitochondrial apoptosis in spinous and basal layer cells [60]. The cyclosporine A-induced proliferation of gingival epithelium is site-specific and cell-layer-specific (significant in the rete peg area) [60], potentially explaining the variability in study results. Previous research also reported the enhancement of GEC proliferation under the stimulation of cyclosporine [61]. Therefore, a comprehensive perspective suggests that cyclosporine promotes GEC renewal through both apoptosis and proliferation.

The level of TGF-β1 in the gingival cervical fluid of periodontitis patients is significantly higher than that of healthy people. TGF-β1 can activate Smad2 and Erk/Akt pathways through GEC receptors, leading to mitochondria-dependent apoptosis [62]. This partially explains how inflammation destroys the epithelial barrier while clearing bacteria. Also, GECs in diabetic periodontitis patients undergo apoptosis due to oxidative stress induced by high glucose, and PINK1-mediated mitophagy can attenuate this damage [63]. The downregulation of mitophagy is considered to be a predisposing factor for diabetes and its various complications. More mechanisms will be discussed later in this review.

As mentioned in the second chapter, *P. gingivalis* has an interesting interaction with GECs. *P. gingivalis* can colonize and replicate within the GECs and resist host clearance through a variety of mechanisms, such as anti-apoptosis mechanisms and by blocking oxidative stress, to ensure its long-term successful survival. Studies have shown that *P. gingivalis* inhibits mitochondrial apoptosis in GECs by activating the JAK/Stat/Akt pathway, inactivating the pro-apoptotic protein Bad and blocking the activation of caspase-3 [64,65]. The virulence factors secreted by *P. gingivalis* also play a role in preventing the apoptosis of host cells. Gingipain addin peptide A44 translocates to mitochondria in the early stage of infection [43]. *P. gingivalis* nucleoside diphosphate kinase Ndk upregulates P2 × 7/NADPH-oxidase (NOX) signaling pathway and inhibits oxidative stress induced by eATP, bypassing the clearance of eATP/NOX2-ROS-antimicrobial defense system [53,66].

#### 4.1.2. Gingival Fibroblasts

Human gingival fibroblasts (hGFs) account for about 65% of the cellular components of gingival connective tissue. HGFs are primarily responsible for the synthesis, degradation and remodeling of the extracellular matrix. Recent studies also show that HGFs act as a sentinel cell of innate immunity, responding to pathogens or trauma by secreting cytokines, chemokines, etc. However, excessive hGF activation caused by periodontal dysbiosis may also promote inflammation and bone destruction [54]. Mitochondria affect hGF homeostasis maintenance, apoptosis, and inflammatory response in many ways.

Periodontitis or periodontal pathogens damage mitochondria and induce oxidative stress in hGFs. In total, 60% of gingival cells from periodontitis patients show abnormal mitochondrial morphology, accompanied by decreased MMP and cellular oxygen consumption, increased ROS, and reduced mtDNA copy number. Moreover, mtDNA sequencing found only 14 mutations in gingival tissue but not in blood samples of periodontitis patients [6,40,67]. In vitro, *P. gingivalis*-LPS stimulates hGFs to produce more mitochondrial ROS before inflammatory factor expression [68]. Mitochondrial targeting exogenous antioxidant (mito-TEMPO) or manganese superoxide dismutase (MnSOD) transfection can inhibit mitochondrial ROS generation, and prevent the increase of IL-1, IL-6 and TNF-α [40,68]. Of great significance for dental clinical operations, dental resin composites (mainly TEGDMA components), blue light irradiation (for resin restoration, bleaching, etc., within 5 min) have also been shown to cause oxidative stress in hGFs by inducing mitochondrial ROS generation [69,70].

Mitochondria are involved in the drug- or infection-induced apoptosis of hGFs. Sodium nitroprusside/nitric oxide regulates the Bcl-2 family and activates JNK to induce hGF apoptosis through a mitochondrial-mediated pathway [71]. Cyclosporine can induce ER stress in hGFs and interfere with the expression of mitochondrial pro-/anti-apoptotic factors [72]. Other studies have shown that infection with periodontal pathogens *F. nucleatum* and *P. gingivalis* alters the expression levels of mitochondria-ER contact-related genes (such as Mfn1/2, PINK1, etc.) in hGFs [73]. Considering that ER stress can upregulate pro-apoptotic factors through mitochondria-ER communication [74], this interactive mechanism may have implications for periodontal tissue destruction.

### 4.2. Periodontal Ligament

#### 4.2.1. Periodontal Ligament Cells

Periodontal ligament cells (PDLCs), also known as periodontal ligament fibroblasts (PDLFs), are the most abundant cells in the periodontal ligament. PDLCs play an essential role in the development of periodontitis through inflammatory response, tissue repair, and periodontal regeneration [75]. Mitochondria are pivotal in the regulation of oxidative metabolism and the management of oxidative stress in PDLCs.

Melatonin, an amine hormone and antioxidant, is reported to have osteogenic and osteoprotective effects. The pharmacological concentration of melatonin (1 μM) was found to increase mitochondrial oxidative metabolism in hPDLCs in vitro, as indicated by increased ATP production and the upregulation of mitochondrial outer membrane translocase 20 (TOMM20), leading to the increased osteogenic activity [76]. However, other research groups have found that physiological concentrations of melatonin (1 pM-10 nM) can disturb mitochondrial function and inhibit the osteogenic differentiation of periodontal ligament stem cells [77].

The apoptosis of PDLCs induced by oxidative stress is one of the major pathogenic mechanisms of periodontitis. Numerous manifestations of mitochondria damage have been observed in hPDLCs under OS state: increased ROS, decreased MMP and ATP production, and enhanced fission [78]. The antioxidant drug madecassic acid can rescue these damages [79]. In addition, the mechanism of hPDLCs pyroptosis is also related to mitochondria. LPS induces pyroptosis of hPDLCs, while ginsenoside (Rg1) can downregulate the expression of pyroptosis-related proteins NLRP3, ASC, caspase-1, and rescue mitochondrial function, showing application potential in the treatment of periodontitis [80].

#### 4.2.2. Periodontal Ligament Stem Cells

Periodontal ligament stem cells (PDLSCs) are considered as the main functional stem cells responsible for the regeneration of alveolar bone, the periodontal ligament, and cementum, making them a highly promising source for tissue engineering. However, the limited success of current regenerative therapy primarily stems from the low survival rate and differentiation capacity of transplanted cells, which can be attributed to local oxidative stress and chronic inflammation at the recipient site [81,82]. Mitochondria play a key regulatory role in PDLSC differentiation and the occurrence of apoptosis under oxidative stress.

Mitochondrial dynamics influence stem cell activities such as self-renewal, proliferation, differentiation, and apoptosis. H_2_O_2_-induced oxidative stress raises ROS level and apoptosis in PDLSCs. Antioxidant curcumin and anti-aging protein Klotho maintain mitochondrial function, reduce ROS, and promote osteogenic differentiation via the PI3K/AKT/FoxO1 and Erk1/2 pathways [81,82]. Other factors have also been shown to affect the osteogenic differentiation of PDLSCs through mitochondria. At a physiological level, melatonin reduces ATP, increases ROS, and inhibits PDLSC osteogenic differentiation [77]. Magnetic stimulation promotes PDLSC proliferation and induces osteogenic differentiation through mitochondria in a time-dependent manner [83].

Oxidative stress promotes PDLSC apoptosis through various mechanisms. Generally, mitochondrial fusion and autophagy protect stem cells from apoptosis, while fission increases their vulnerability to stress and promotes apoptosis [19]. Cobalt chloride (CoCl_2_) upregulates the expression of Drp1 in PDLSCs, promotes excessive mitochondrial fission, and induces OS and apoptosis of PDLSCs [84]. Advanced glycation end products (AGEs) induce endogenous ROS formation in PDLCs and PDLSCs and initiate mitochondria-mediated apoptosis through JNK pathway. This partly explains the connection between diabetes and periodontitis [49,85]. The antioxidant, moringin, inhibits OS-induced mitochondrial dysfunction and apoptosis in PDLSCs by downregulating PINK and upregulating Bcl-2 [86].

### 4.3. Alveolar Bone

Osteoclasts (OCs) and osteoblasts (OBs) mediate bone resorption and formation, respectively, and these processes require significant energy expenditure in vivo. Recent research has revealed the substantial presence of mitochondria in mature OCs and OBs, highlighting the close involvement of mitochondria in the maturation, metabolism, and proliferation of these cells. Mitochondria play critical roles in bioenergy generation, signal transduction, dynamics, mitophagy, and other essential aspects of OC and OB function.

#### 4.3.1. Osteoclasts

OCs originate from blood monocyte–macrophages (also known as osteoclast precursor cells), which differentiate and fuse to form multinucleated cells under the induction of cytokines, such as M-CSF and RANKL, and then further form terminally differentiated bone resorptic cells [87]. The production and increased activity of OCs has been linked to a number of diseases, such as osteoporosis, rheumatoid arthritis, and periodontitis, in which mitochondria play an essential regulatory role. The inhibitory effects of estrogen, PRF/BCP, and various antioxidants such as hydroxytyrotol (HT) and coenzyme Q10 on OC differentiation have all been shown to be associated with mitochondria-mediated apoptosis [88,89,90,91]. Generally, oxidative stress promotes the differentiation of peripheral blood monocytes into OCs. The antioxidant catechin EGCG and other polyphenolic compounds can inhibit mitophagy and reduce ROS, thereby inhibiting OC differentiation [92,93]. On the contrary, doxorubicin, a chemotherapeutic drug that increases the risk of bone damage in patients, enhances OC activity by increasing mitochondrial ROS and inducing excessive autophagy [94]. Interestingly, in addition to the effects of oxidative stress on OC production, mitochondrial outer membrane protein Mfn2 can also regulate OC differentiation and activity by affecting intracellular calcium signaling [95]. These studies promoted the importance of previously underappreciated mitochondria in OC generation.

Studying the metabolic requirements and bioenergetics of osteoclast (OC) generation provides insights into its biological properties during bone resorption. Although the oxidative phosphorylation of glucose is the major bioenergetic pathway in OC differentiation, the aerobic glycolysis of the generated lactate is also indispensable. The mutation of Glut1, a glucose transporter, reduces OC differentiation in vitro and leads to osteopetrosis in female mice [96]. OC differentiation also requires mitochondrial long-chain fatty acid oxidation. Inhibiting Cpt2, a key enzyme in this pathway, reduces the formation of multinucleated OCs and increases cancellous bone in mice [97]. These effects may be attributed to the acidic microenvironment in mature OCs, which promotes hydroxyapatite ionization, the secretion of matrix-degrading enzymes, and cell migration.

#### 4.3.2. Osteoblasts

OBs can be derived from chondrocytes, mesenchymal stem cells, bone-lining cells and special craniofacial fibroblasts (such as periodontal ligament stem cells). The enriched rough ER, Golgi apparatus and mitochondria in OBs guarantee their function of synthesizing collagen and depositing hydroxyapatite [96,97,98,99]. The impact of mitochondria on OBs can be categorized into two aspects: the regulation of their differentiation, maturation, and metabolism; and their association with programmed cell death.

The process of OB differentiation exhibits a particular metabolic pattern. In aerobic conditions, glucose consumption and lactate production significantly increase during OB differentiation, while oxygen consumption decreases. Both OBs and OCs show a tendency to glycolysis during their differentiation, and aerobic glycolysis can account for approximately 80% of the total ATP production in OBs [100]. The knockdown of Me2 significantly reduced glycolytic flux and impaired OB proliferation and differentiation, underscoring the link between the aerobic glycolysis of OBs and mitochondria [100]. The disruption of this energy metabolism pattern may lead to impaired OB formation and function. An in vitro high-glucose environment (30.5 mM) changes the OB metabolic mode, causing it to use both mitochondrial respiration and glycolysis, favoring the former, and OB differentiation and mineralization were inhibited [50]. However, another research group reported that high glucose (11.1 mM) promoted OB differentiation and mineralization, did not affect its proliferation, but reduced OB migration ability and chemotaxis, accompanied by decreased mitochondrial biogenesis [101]. These conflicting results stem from differences in in vitro OB culture conditions and require comprehensive interpretation.

Mitochondria influence OB differentiation through biogenesis, quality control, and dynamics. PGC-1α regulates mitochondrial biogenesis, and its upregulation by polyphenolic compounds or AdipoRs agonists promotes OB differentiation [24,25]. PINK1 is essential for mitochondrial quality control, and its knockdown impairs OB differentiation and calcium metabolism [102], indicating that PINK1 activation is necessary for OB differentiation. Recent studies have shown that there is a substantial amount of mitochondrial component in the bone matrix and that mature OBs promote the differentiation of pre-osteoblasts by secreting mitochondria and mitochondria-derived vesicles into the extracellular space. In addition, mitochondrial fission is increased during OB differentiation. The knockdown of OPA1 increases mitochondrial fission and can promote osteogenesis [103].

The programmed death of OBs can be induced by a variety of factors that are closely related to mitochondria, such as hypoxia, oxidative stress, and abnormal calcium metabolism. Hypoxia can induce mitochondrial dysfunction and stimulate mitophagy and apoptosis in OBs, leading to periapical bone loss [51]. Oxidative-stress-induced apoptosis plays a crucial role in the occurrence and development of osteoporosis. Various antioxidants rescue OBs from oxidative stress by targeting mitochondrial functions, such as hydroxytyrosyl [104] and proanthocyanidins [105]. The pharmacological inhibition or knockdown of Drp1 can also rescue oxidative-stress-induced OB dysfunction [106]. Moreover, abnormal calcium metabolism can also promote OB apoptosis. Melatonin upregulates STIM1 to increase cytoplasmic calcium level, and induces mitochondrial apoptosis of OBs through ERK pathway [107].

### 4.4. Cementum

Cementum is a thin layer of mineralized connective tissue deposited on the surface of the tooth root by cementoblasts (CBs). Cementum is involved in the occurrence and repair of periodontal lesions; therefore, the damage and dysfunction of CBs can exacerbate the development of periodontitis. Our research group has long been concerned about cementogenesis and CB function, clarifying the important role of Osterix in CB differentiation [108,109]. Furthermore, we have elucidated multiple molecular mechanisms underlying the inhibition of CB mineralization during periodontitis, including identifying the positive regulatory genes, CXXC5 and miR-181b-5p [110,111,112], and negative regulatory genes, CKIP-1 and miR-155-3p, of CB mineralization [113,114]. Additionally, we have reported on the enhancing effect of M2 macrophages on CB mineralization [115,116].

Studies have shown that the formation and mineralization of CBs depends on normal mitochondrial function. Hypoxia induced by CoCl_2_ causes abnormal mitochondrial morphology and decreases biosynthesis by downregulating PGC-1α expression in CBs through p38 and Erk1/2 signaling pathways, thereby inhibiting CB mineralization [22]. In addition, other studies have focused on the cementogenic differentiation of PDLSCs under inflammatory conditions. LncRNA GACAT2 can directly bind to and activate the mitochondria-related protein pyruvate kinase PKM1/2, leading to the translocation of PKM2 to mitochondria and promoting the cementogenic differentiation of PDLSCs [55].

### 4.5. Others

#### 4.5.1. Macrophages

The conversion of macrophages to the pro-inflammatory M1 phenotype is strongly associated with the progression of periodontitis, with increased permeability of the mitochondrial membrane and the excessive release of ROS being the direct triggers [117]. The application of macrophage-targeted calcium nanoparticles to regulate MPTP, or the application of mitophagy activators such as resveratrol, can reduce ROS release and alleviate periodontal inflammation [39]. On the other hand, some studies have also reported that molybdenum enhances macrophage M2 polarization to promote periodontal wound healing by enhancing mitochondrial function and regulating oxidative metabolism [118]. Moreover, in addition to its antibacterial effects, it is reported that methylene-blue-mediated photodynamic therapy alleviates inflammation by inducing mitochondria-mediated apoptosis in macrophages [119].

#### 4.5.2. Neutrophils

In periodontal tissue, neutrophils respond to the formation of pathogenic biofilms by producing bactericidal ROS. However, the excess release of ROS can cause tissue damage and exacerbate inflammation. Therefore, neutrophils activate a specialized antioxidant system Nrf2 signaling pathway. Nrf2 not only participates in mitochondrial biogenesis, but also regulates the expression of antioxidant enzymes, thereby protecting the human body from oxidative stress [120]. The levels of Nrf2 and downstream antioxidant enzymes are significantly downregulated in neutrophils of patients with severe periodontitis [121].

## 5. The Relationship between Mitochondria and Periodontitis-Related Systemic Diseases

**Figure 2:**
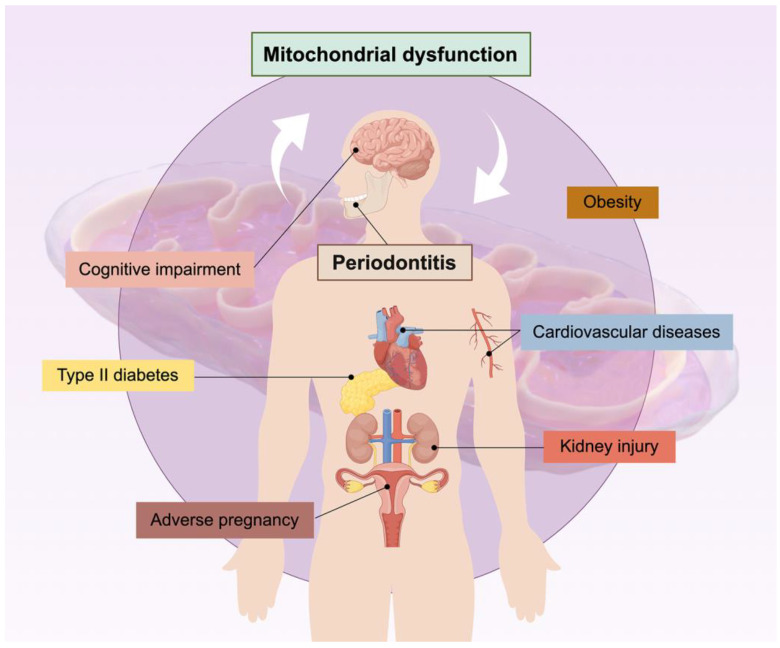
Periodontitis is associated with a variety of systemic diseases through the shared pathology of mitochondrial dysfunction. This figure was generated by Figdraw (version 2.0).

Periodontitis is associated with the occurrence, progression, and prognosis of a variety of systemic diseases (Figure 2), and it is the focus of a large body of evidence-based medical research. This chapter focuses on these systemic diseases and elaborates on the role of mitochondria dysfunction in the association of periodontitis with these diseases.

### 5.1. Type II Diabetes Mellitus

Type 2 diabetes mellitus (T2D) is a metabolic disease that causes a global burden. Mitochondria are involved in the occurrence and development of T2D [7]. About a decade ago, the bidirectional relationship between chronic periodontitis (CP) and T2D began to attract academic attention [122]. CP and T2D may share a common pathological process through mitochondrial dysfunction and oxidative stress [12].

Numerous studies have implicated that the mechanism by which T2D causes or exacerbates periodontal tissue damage is mitochondria-related. Hyperglycemia leads to increased formation of AGEs, which are associated with the pathogenesis of both CP and T2D. Experiments confirmed that AGEs induce endogenous ROS in PDLCs and induce autophagy, activating the downstream JNK pathway to initiate mitochondria-mediated apoptosis [49,85]. High glucose inhibits OB mineralization and renders OB energy metabolism towards an early differentiation state [50]. High glucose also induces the early apoptosis of GECs, which can be attenuated by PINK-mediated mitophagy [54].

In vivo research further broadens our knowledge of how mitochondria play a role in the relationship between T2D and periodontitis. In Wistar rats, the CP-T2D group showed more bone loss and periodontal cell apoptosis than the CP group [123], along with more severe mitochondrial dysfunction. A multiple regression analysis revealed a close association between these mitochondrial events and bone loss in diabetic periodontitis [124]. In addition, unbalanced oxidative stress emerged as a key link in T2D-CP. CP led to significant local tissue oxidative damage, while T2D resulted in systemic oxidative damage and reduced antioxidant capacity in rats [123]. The co-occurrence of the two can synergistically aggravate local and systemic oxidative damage, which partly explains the more severe periodontal destruction in CP-T2D.

Oxidative stress is a critical factor in the context of T2D-CP, as discussed earlier. Several studies have focused on antioxidant-based interventions for the treatment of T2D-CP. The transcription factor Nrf2 plays a key role in cellular defense against oxidative stress and is closely related to mitochondrial biogenesis. Nrf2 expression was significantly reduced in T2D-CP comorbid rats [123]. However, conflicting results have also been reported, with the upregulated expression of Nrf2 observed in the peripheral blood mononuclear cells of T2D-CP patients [12]. While the level of expression of Nrf2 is still debated, in vitro and in vivo experiments have demonstrated the beneficial effects of its upregulation. Xanthanol (BCI) inhibits oxidative stress in hGECs cultured with high glucose/LPS and mitigates periodontal damage in T2D-CP rats by promoting pNrf2 nuclear translocation and upregulating Nrf2 [125].

### 5.2. Cognitive Impairment

The role of mitochondria in neurodegenerative diseases has long attracted attention. High oxygen consumption and high levels of polyunsaturated fatty acids in the brain determine its susceptibility to oxidative damage [126]. Epidemiological evidence has demonstrated the association between periodontitis and cognitive impairment [9,127]. Notably, *P. gingivalis*, a major pathogen of periodontitis, contributes to neuroinflammation and neurodegenerative diseases such as Alzheimer’s disease (AD) [41,128]. Mitochondrial dysfunction may serve as a potential mechanism underlying the comorbidity observed between periodontitis and cognitive impairment.

The accumulation of methylmalonic acid (MMA) in tissues is associated with mitochondrial energy imbalance and ATP synthesis disorders, serving as a biomarker of mitochondrial dysfunction [13]. A mediation analysis study showed that circulating MMA levels mediated the association between periodontal status indicators (PD, AL) and cognitive function (5.8–11.7%) [13], which confirmed that mitochondrial dysfunction played a mediating role between periodontitis and cognitive impairment in the elderly from an epidemiological perspective. On the other hand, treatment with *P. gingivalis*-LPS in vitro in human neuroblastoma cells had an overall negative effect on mitochondria function through TLR4 activation. The TLR4-specific inhibitor CLI-095 can restore mitochondrial damage, which may be used as a therapeutic approach for *P. gingivalis*-LPS-induced neuroinflammation [41].

### 5.3. Obesity

Obesity refers to excessive body fat accumulation that leads to health problems [7]. Epidemiological studies have demonstrated the association between obesity and the incidence of periodontitis [14,129]. This is mainly due to impaired immune response and chronic inflammation in obese patients [23], as well as oxidative stress, which is linked to mitochondrial function [14].

Obese patients have higher levels of pro-inflammatory factors (such as leptin, TNF-α, IL-1β, etc.) and lower levels of anti-inflammatory factors (such as adiponectin) in serum and gingival crevicular fluid, implying enhanced systemic and local inflammation [14]. Case–control studies have also shown higher levels of periodontal oxidative stress in obese patients compared to normal-weight patients [15]. For obese patients with periodontitis, although non-surgical periodontal therapy alone improved leukocyte homeostasis, the inhibition of systemic inflammation and oxidative stress was not significant. When combined with diet therapy, it can effectively reduce TNF and the total number of ROS, as well as increasing SOD activity [130].

Conversely, there is limited research on the effects of periodontitis on obesity, but it is suggested that *P. gingivalis* and its virulence factors can induce inflammation and adipocyte apoptosis by affecting mitochondrial function. *P. gingivalis* causes the inflammation and dysfunction of adipocytes in vivo and in vitro by increasing the levels of TNF-α, IL-6, and iNOS, and reducing the expression of PGC-1α and adiponectin, while Kavain has anti-inflammatory and antioxidant effects on adipocytes through the activation of PGC-1α [23]. *P. gingivalis*-LPS initiates the mitochondria-mediated apoptosis of adipocytes by enhancing leptin and reducing adiponectin expression in rats [45].

### 5.4. Cardiovascular Diseases

Normal mitochondrial function is essential for the cardiovascular system. For example, mitochondrial fission is necessary for MQC and cell homeostasis, but this process is usually abnormally activated in cardiovascular diseases, directly leading to the development of these diseases [131]. In addition, mitochondrial quality control is crucial for cardiac aging [132].

Overall, people with periodontitis have a higher risk of developing multiple cardiovascular diseases. Possible mechanisms include bacteremia, systemic inflammatory state, and increased ROS in neutrophils [10]. *P. gingivalis*, the major pathogen of periodontitis, is reported to accelerate the progression of atherosclerosis (AS) through mitochondrial dysfunction [11]. However, there is insufficient evidence on the effect of cardiovascular disease on periodontitis.

Studies have shown that *P. gingivalis* can cause mitochondrial fission and dysfunction in vascular endothelial cells by upregulating the phosphorylation of Drp1 and facilitating its translocation to mitochondria, and the Drp1 inhibitor Mdivi-1 can block such effects of *P. gingivalis* [11]. Besides fission, mitochondria also affect the occurrence of periodontitis cardiovascular disease by regulating oxidative stress. *P. gingivalis* dihydroceramide, a virulence factor that provokes inflammatory response in hGFs, increases ROS and mitochondrial-dependent apoptosis in endothelial cells [133]. In patients with periodontitis, there is an increase in oxidative stress in peripheral blood mononuclear cells and polymorphonuclear leukocytes [42,134], which may lead to AS and other cardiovascular complications.

### 5.5. Other Systemic Diseases

#### 5.5.1. Adverse Pregnancy

Periodontal disease is associated with numerous pregnancy complications, and one of the most significant concerns is low birth weight (LBW). In women with periodontitis, bacteria and their byproducts in infected periodontal tissues can enter the fetoplacental unit through circulation, leading to inflammation and cell death [135]. *A. actinomycetemcomitos*-LPS induced the apoptosis of human placental trophoblastic cells in vitro through the mitochondria-dependent pathway, and an in vivo TUNEL assay confirmed that *A. actinomycetemcomitos*-LPS treatment increased the incidence of apoptosis in rat placental cells [46]. This mechanism may contribute to the development of LBW in relation to periodontitis.

#### 5.5.2. Kidney Injury

Mitochondrial dysfunction is reported to trigger kidney disorders, which are another systemic disease associated with periodontitis [7]. Rats with periodontitis experienced renal structural damage, elevated levels of oxidative stress, mitochondrial structural damage, ROS accumulation, and decreased expressions of SIRT1 and PGC-1α. However, no significant impairment of renal function was detected. The antioxidant resveratrol can prevent kidney injury and mitigate mitochondrial dysfunction in rats with periodontitis [136].

## 6. Mitochondria-Targeted Therapy

In light of the diverse mechanisms underlying mitochondrial dysfunction, several therapeutic regulators have been studied. Some of these hold promise for mitochondria-targeted therapy, while others aid in a deeper understanding of the pathogenic mechanisms of the diseases. Table 1 summarizes the therapeutic regulators that have demonstrated therapeutic potential in periodontitis and its associated systemic diseases.

Antioxidants constitute a major category among these therapeutic modulators and have garnered significant attention in the field of mitochondria-targeted therapy. The mechanisms of antioxidants can be categorized as follows: enhancing mitochondrial respiration, promoting mitochondrial biogenesis, regulating mitophagy, and alleviating oxidative stress by scavenging ROS. Through these mechanisms, antioxidants exert therapeutic effects by alleviating tissue destruction, reducing inflammation, and promoting regeneration. Most importantly, antioxidants may serve as a mediator in alleviating systemic oxidative stress and inflammatory states induced by periodontitis-associated systemic diseases [7,136]. Mitochondria-targeted therapy can serve as an adjunctive approach for periodontitis and its associated systemic diseases. However, as an infectious disease, the primary treatment for periodontitis remains the control of bacterial infection. Additionally, it would be intriguing to investigate the impact of combining preventive treatments, such as ozone and photobiomodulation, with mitochondrial therapy on oral microbiota in order to gain insights into their synergistic effects [137,138].

**Table 1:** Therapeutic regulators of mitochondrial function in cells associated with periodontitis and systemic diseases.

Type	Therapeutic Regulator	Cell Type	Function	Mechanism	Ref.
Antioxidant	CoQ10	Fibroblast	Improve mitochondria biogenesis	Activate PGC-1α and TFAM	[26]
		OC	Inhibit RANKL-induced osteoclastogenesis	Regulate mitochondrial apoptosis and oxidative stress	[91]
	Resveratrol	MØ	Anti-inflammation	Induce PINK1-mediated mitophagy	[39]
		OB	Induce OB differentiation	Stimulate mitochondrial respiration and biogenesis via PGC-1α upregulation	[24]
		Renal tissue cell	Protect renal tissue from periodontitis-induced damage	Prevent mitochondrial dysfunction	[136]
	Hydroxytyrosol	Pre-OB; OC	Protect pre-OB from OS damage, inhibit OC differentiation, attenuate bone loss in periodontitis mice	Modulate mitochondria function, ERK and JNK signaling pathways	[90]
		OB	Prevent OS-induced OB apoptosis	Decrease OPA1 cleavage and increase AKT and GSK3β phosphorylation to inhibit OS-induced mitochondrial dysfunction	[104]
	EGCG	OC	Inhibit OC differentiation	Modulate mitophagy through AKT and p38 signaling pathways; block RANK-RANKL binding	[92]
		OB	Induce OB differentiation	Stimulate mitochondrial respiration and biogenesis via PGC-1α upregulation	[24]
	Polyphenolic Compounds (tannic acid, gallic acid, and ellagic acid)	OC	Inhibit OC differentiation	Inactivate Akt to suppress autophagy; downregulate ROS, cellular Ca^2+^ and MMP	[93]
	Isovitexin	OB	Promote OB differentiation	Upregulate PGC-1α via AdipoRs to induce OxPhos and ATP production	[25]
	Proanthocyanidins	OB	Ameliorate OB oxidative stress and mitochondrial dysfunction	Activate Nrf2 signaling	[105]
	Mito-TEMPO; MnSOD	hGF	Prevent LPS-induced pro-inflammatory response in HGF	Inhibit mtROS to downregulate IL-1β, IL-6 and TNF-α, inhibit p38, c-Jun N-terminal kinase and inhibitor of nuclear factor-κB kinase	[68]
	Madecassic acid	hPDLC	Protect HPDLC from oxidative stress and apoptosis	Reduce ROS and maintain MMP; downregulate Bax, upregulate Bcl-2 and Bcl-xL	[79]
	Moringin	hPDLSC	Inhibit OS-induced mitochondria dysfunction	Downregulate PINK1 to prevent mitophagy, downregulate Bax and caspases, upregulate Bal2L12 and MCL1 to inactivate apoptosis	[86]
	Curcumin	hPDLSC	Reduce apoptosis and promote osteogenesis in HPDLSC	Reduce ROS, modulate Erk1/2 signaling pathway	[81]
	Baicalein	hGEC	Prevent HGEC oxidative stress and reduce bone loss in CPDM mice	Modulate Nrf2 signaling pathway	[125]
Hormone, antioxidant	Melatonin	OB	Induce OB mitochondrial apoptosis	Upregulate STIM1/cytosolic calcium elevation/ERK pathway	[107]
		hGF	Attenuate ER stress, fibrosis and mitochondrial apoptosis markers	Decrease VEGF; significantly decrease CHOP, GRP78, and XBP1s; significantly increase cytochrome c, significantly decrease cyclophilin D	[72]
		hPDLC	Promote HPDLC osteogenesis (pharmacological concentration)	Upregulate TOM20 to enhance mitochondrial function	[76]
		hPDLSC	Suppress HPDLSC osteogenic differentiation and alter mitochondrial function (physiological concentration)	Downregulate OPN and OCN; decrease ATP, increase ROS and NAD+/NADH ratio	[77]
Hormone	Estrogen	OC	Increase OC precursor mitochondria-induced apoptosis, decrease OC number	Attenuate mitochondria oxidative phosphorylation and ATP production, promote OC precursor apoptosis through Bak/Bax	[88]
Mitochondrial fission inhibitor	Mdivi-1	Endothelial cell	Inhibit Pg-induced mitochondrial fission and dysfunction	Inhibit assembly and activity of Drp1	[11]
Mitochondrial fusion promoter	M1	OB	Impair osteogenesis	Upregulate OPA1 expression	[103]
Ferroptosis inhibitor	Fer-1	hGF	Attenuate Pg-LPS-induced inflammation	-	[47]
ER stress inhibitor	4PBA	hGF	Attenuate ER stress, fibrosis and mitochondrial apoptosis markers	Decrease CTGF, VEGF, TGFβ, and α-SMA; significantly decrease ER stress markers, CHOP, GRP78, XBP1, XBP1s; significantly increase cytochrome c, decrease both Bcl-2 and cyclophilin D	[72]
Hypoxia mimetic	CoCl_2_	hPDLSC	Induce HPDLSC apoptosis	Increase ROS and Drp1 to promote mitochondrial fission	[84]
		CB	Inhibits CB mineralization and mitochondrial biogenesis	Downregulate PGC-1α via p38 and Erk1/2 signaling pathways	[22]
Cardiovascular drug	Simvastatin	OB	Inhibit mitophagy-related OB apoptosis and alleviate bone resorption	Alleviated hypoxia-induced mitochondrial dysfunction, mitophagy and apoptosis	[51]
		hGF	Attenuate ER stress, fibrosis and mitochondrial apoptosis markers	Decrease VEGF, CTGF, TGFβ; significantly decrease CHOP, GRP78, and XBP1s; significantly increase cytochrome c, decrease cyclophilin D	[72]
	Nitric oxide (Sodium nitroprusside)	hGF	Induce HGF apoptosis	Induce the loss of MMP, increase Bax/Bcl-2 ratio and activate JNK and caspases	[71]
Chemotherapy drug	Doxorubicin	OC	Induce bone loss by increasing OC autophagy	Increase mitochondrial ROS/TRPML1/TFEB axis in OC	[94]
	Ginsenoside Rg1	hPDLC	Alleviate LPS-induced HPDLC pyroptosis	Upregulate AMPK-dependent Drp-1 phosphorylation to inhibit mitochondrial fission; reduce NLRP3, ASC, caspase-1 and GSDMD-NT	[80]
Immunosuppressant	Cyclosporine A	hGEC	Enhance mitochondrial apoptosis in gingival keratinocytes	Upregulate Bax, AIF, caspase-3/9 expression	[59]
			Inhibit mitochondrial apoptosis in spinous and basal layer cells	Upregulate Bcl-2, downregulate caspase-3	[60]
		hGF	Induce ER stress	Interference with mitochondrial pro- and anti-apoptotic factors	[72]
Cell component	Myo-EV	OC	Suppress OC formation and mitochondrial energy metabolism	Suppress oxygen consumption and mitochondria biogenesis	[139]
	Mitochondria and MDV	OB	Promote osteogenesis	Elevate BMP signaling in osteoprogenitor cell	[103]
Peptide	7R-ETGE	OC	Attenuate RANKL-dependent osteoclastogenesis and bone destruction, decrease ROS level	Prevent Keap1 from binding to Nrf2, induce Nrf2 nuclear translocation and Nrf2-dependent cytoprotective enzyme expression	[140]
Dental material	PRF/BCP	OC	Impair OC differentiation, promote mitochondria-induced apoptosis	Inhibit NF-kappa B and Bcl-2/xL, promote caspase-3 and Bax levels	[89]
	TEGDMA	hGF	Induce oxidative stress and mitochondrial dysfunction in HGF	Increase ROS, aberrant mitochondrial morphology and functions	[69]
	Molybdenum	MØ	Promote periodontal wound healing	Effect mitochondrial structure, MMP and promotes metabolic shift from glycolysis toward mitochondrial OXPHOS	[118]
Physical treatment	Magnetic stimulation	hPDLSC	Induce proliferation and osteogenesis in HPDLSC	Promote Erk phosphorylation; Alter mitochondrial respiration	[83]
	Methylene blue-mediated photodynamic therapy	MØ	Alleviate bone loss in periodontitis	Induce mitochondrial-mediated MØ apoptosis	[119]

**Figure 3:**
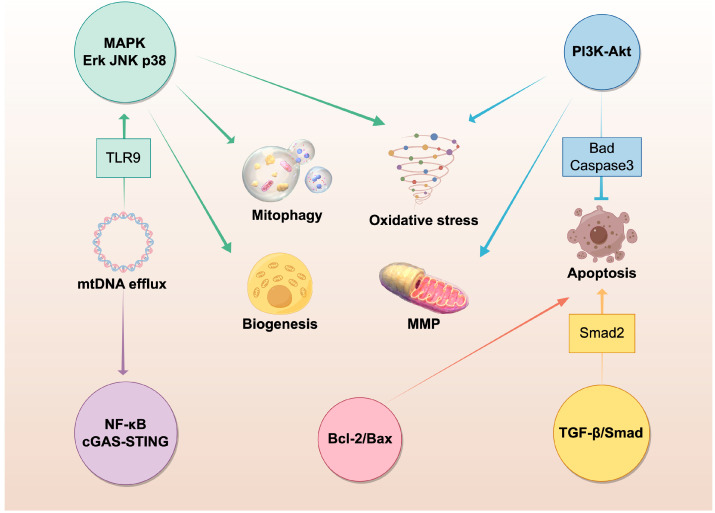
Mitochondrial function is regulated through multiple pathways in the patho-mechanism of periodontitis and systemic diseases. Pointed arrows: promoting effect; bar-headed arrow: inhibitory effect. The figure was generated by Figdraw (version 2.0).

Moreover, elucidating the regulatory role of signaling pathways in mitochondria-targeted therapy is significant. Figure 3 summarizes the multiple signaling pathways pertaining to mitochondrial function that are of interest in the research of periodontitis and its associated systemic diseases, as highlighted in this review. Further investigation is warranted to explore the involvement of these key signaling pathways in the pathogenesis of periodontitis and its associated systemic diseases in the context of mitochondrial dysfunction.

## 7. Conclusions

Like many infectious diseases, although periodontitis is provoked by bacteria colonizing the tooth surface and gingival sulcus, the host response plays an indispensable role in the devastation of connective tissue and bone. Among these responses, mitochondrial function exhibits rapid and pronounced alterations, sometimes preceding the inflammatory response itself [68]. Fluctuations in mitochondrial function can be seen as critical signaling events and one of the most direct clues for when cells encounter external stimuli. The multifaceted manifestations of mitochondrial dysfunction, such as reduced biosynthesis, disrupted dynamics, dysregulated mitophagy, abnormal metabolism, mtDNA efflux, oxidative stress, and mitochondria-mediated apoptosis, are typically observed in the various tissues and cells involved in the occurrence and progression of periodontitis. Understanding these mechanisms and adopting mitochondrial-targeted therapies as adjuncts to conventional mechanical debridement hold promise as new concepts for periodontitis control.

Moreover, mitochondrial dysfunction is an important pathological mechanism linking periodontitis with associated systemic diseases. For example, bacteremia caused by pathogens and their virulence factors entering the bloodstream can lead to mitochondrial dysfunction in distant target cells, fostering the development of systemic diseases. Conversely, systemic diseases resulting in oxidative stress can exacerbate local periodontal damage. A deeper understanding of mitochondrial dysfunction not only elucidates the mechanisms by which cardiovascular drugs, chemotherapy drugs, and others can induce periodontitis, but also presents new options like antioxidants for comorbid patients such as T2D-CP and AS-CP.

Indeed, current mitochondrial-targeted therapies exhibit certain limitations, including a dearth of data from animal or human experiments, inconsistent efficacy, and a need for more rigorous administration and dosing, which need further improvement. Moreover, this review also has its own limitations, such as the incomplete retrieval of literature and reporting biases. However, the overall perspective and latest evidence provided in this review underscore the paramount role of mitochondria in the pathogenesis and therapy of periodontitis and associated systemic diseases, propelling future research towards unravelling the intricacies of periodontitis and its interplay with systemic diseases.

## Data Availability

Not applicable.
